# Quantitative Analysis of the Membrane Affinity of Local Anesthetics Using a Model Cell Membrane

**DOI:** 10.3390/membranes11080579

**Published:** 2021-07-30

**Authors:** Wanjae Choi, Hyunil Ryu, Ahmed Fuwad, Seulmini Goh, Chaoge Zhou, Jiwook Shim, Masahiro Takagi, Soonjo Kwon, Sun Min Kim, Tae-Joon Jeon

**Affiliations:** 1 Department of Biological Engineering, Inha University, Incheon 22212, Korea; choiwj1118@gmail.com (W.C.); hyunil.ryu@gmail.com (H.R.); sm1goh@naver.com (S.G.); zhouchaoge1995@gmail.com (C.Z.); soonjo.kwon@inha.ac.kr (S.K.); 2 Department of Mechanical Engineering, Inha University, Incheon 22212, Korea; ahmedsunny41@gmail.com; 3 Department of Biomedical Engineering, Rowan University, Glassboro, NJ 08028, USA; happysjw@gmail.com; 4 School of Materials Science, Japan Advanced Institute of Science and Technology, 1-1 Asahidai, Nomi 923–1292, Ishikawa, Japan; takagi@jaist.ac.jp; 5 Department of Biological Sciences and Bioengineering, Inha University, Incheon 22212, Korea

**Keywords:** local anesthetics, membrane affinity, lipid bilayer membrane, calorimetric assay, cramicidin A, fibroblast cells

## Abstract

Local anesthesia is a drug that penetrates the nerve cell membrane and binds to the voltage gate sodium channel, inhibiting the membrane potential and neurotransmission. It is mainly used in clinical uses to address the pain of surgical procedures in the local area. Local anesthetics (LAs), however, can be incorporated into the membrane, reducing the thermal stability of the membrane as well as altering membrane properties such as fluidity, permeability, and lipid packing order. The effects of LAs on the membrane are not yet fully understood, despite a number of previous studies. In particular, it is necessary to analyze which is the more dominant factor, the membrane affinity or the structural perturbation of the membrane. To analyze the effects of LAs on the cell membrane and compare the results with those from model membranes, morphological analysis and 50% inhibitory concentration (IC50) measurement of CCD-1064sk (fibroblast, human skin) membranes were carried out for lidocaine (LDC) and tetracaine (TTC), the most popular LAs in clinical use. Furthermore, the membrane affinity of the LAs was quantitatively analyzed using a colorimetric polydiacetylene assay, where the color shift represents their distribution in the membrane. Further, to confirm the membrane affinity and structural effects of the membranes, we performed an electrophysiological study using a model protein (gramicidin A, gA) and measured the channel lifetime of the model protein on the free-standing lipid bilayer according to the concentration of each LA. Our results show that when LAs interact with cell membranes, membrane affinity is a more dominant factor than steric or conformational effects of the membrane.

## 1. Introduction

Local anesthetics (LAs) are amphiphilic compounds that mainly consist of a lipophilic aromatic ring and a hydrophilic amino group connected through an amide or an ester [1]. Local anesthetics are essential drugs, especially in modern medical surgery, to suppress pain, in particular by blocking the transmission of nerve signals [1,2]. The mechanism of pain suppression by LAs is generally related to the deactivation of sodium ion channels, and several studies suggest a direct interaction mechanism between LAs and the binding sites of ion channel proteins causing conformational changes and hence the blockage of voltage-gated cation channels [3,4]. Numerous studies have revealed that in addition to affecting sodium ion channels, LAs affect other functional proteins, such as Na+/K+-ATPase [5,6] glycine receptors and γ-aminobutyric acid receptors [7]. However, this direct mechanism alone cannot fully explain the pharmacological and toxicological effects of LAs.

Recent studies alternatively suggest an indirect interaction mechanism between LAs and ion channel proteins. Due to the amphiphilic nature of LA compounds, they partition into cell membranes and perturb the cell membrane matrix by modifying its physiochemical properties, such as permeability, fluidity, and electrostatic potential, which eventually affect membrane protein functions [8,9,10]. This indirect interaction model explains the mechanism of some local anesthetics and the associated toxic effects caused by LAs such as lidocaine, tetracaine, bupivacaine, and procaine [11,12]. Recently, several approaches were reported to analyze the effects of LAs on model-cell membranes. Weizenmann et al. analyzed the location and orientation of the lipid bilayer of LAs using NMR spectroscopy [13]. Pardo et al. measured the conductance of membranes and confirmed that LAs could alter membrane permeability [14]. Tsuchiya et al. found increased membrane fluidity due to LAs through UV fluorescence polarization measurement [15]. Sugahara et al. investigated changes in the thermal stability of lipid membranes in the presence of LAs using a differential scanning calorimetry (DSC) assay. The addition of LAs to cholesterol-contained liposomes decreases the miscibility of the liquid-ordered phase and liquid-disordered phase as well as the main transition temperature of the membrane [16]. S. Efimova et al. confirmed that LAs change membrane electrostatic potentials through a model membrane experiment using a planar lipid bilayer and gramicidin A (gA) [17]. However, these studies cannot explain the exact mechanism for diverse LAs with different structures.

**Figure 1:**
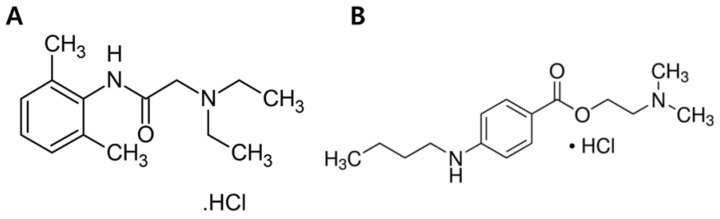
Chemical structure of LAs (**A**) Lidocaine, (**B**) Tetracaine.

In this study, we analyzed and compared the effects of the most commonly used LAs, amide-based lidocaine (LDC) and ester-based tetracaine (TTC), on cell membranes and ion channels (Figure 1). Clinically, both LDC and TTC are applied to human skin fibroblasts and affect the target area; therefore, we analyzed the cytotoxicity of LAs to human skin fibroblast cells, CCD-1064sk, using lactate dehydrogenase (LDH) and Cell Counting Kit-8 (CCK-8) assays. Furthermore, we analyzed the distribution effect of LAs through morphological analysis of normal human skin fibroblast cells using field emission scanning electron microscopy (FESEM). We also analyzed the membrane affinity of LDC and TTC through colorimetric assays using polydiacetylene (PDA) vesicles. PDA vesicles have a similar structure to lipid membranes in which color transition occurs upon external stimuli. Finally, we performed electrophysiological experiments to further analyze the structural effects of LDC and TTC on the cell membrane and their indirect effects on the model cell protein gA. We found that the membrane affinity of LAs is a more crucial factor when interacting with cell membranes in determining their toxicological and pharmacological studies.

## 2. Materials and Methods

### 2.1. Cell Culture

CCD-1064sk cells were purchased from the American Type Culture Collection (ATCC). Initially, the preadipocytes were put into 96-well plates at a density of 10,000 cells per well and cultured at 37 °C in 5% CO_2_ in Iscove’s modified Dulbecco’s medium (IMDM; Gibco, Carlsbad, CA, USA) supplemented with 10% fetal bovine serum (FBS; Gibco, Carlsbad, CA, USA). After two days, the cells reached confluence, and then the cells were treated with LDC (Sigma-Aldrich, St. Louis, MO, USA) and TTC (Sigma-Aldrich, St. Louis, MO, USA) for 24 h.

### 2.2. LDH Assay

Cell viability in the presence of LDC and TTC was analyzed using the LDH assay (EZ-LDH, DoGen, Guro, Korea). Cell viability was investigated in the control and LA-treated groups at various concentrations. Cells were treated with LDC at concentrations of 0.1, 0.3, 0.5, 0.7, 1, 1.3, 1.5, 2, 2.5, and 3 mM. TTC was tested at concentrations of 0.01, 0.1, 0.2, 0.3, 0.45, 0.5, 0.55, 0.6, 0.7, 0.8, 0.9, 1, and 1.5 mM. Since 0.1% DMSO was used as the vehicle for the LAs, control group cell viability was tested with 0.1% DMSO. CCD-1064sk cells were seeded into 96-well plates at a density of 10,000 cells per well. Ten microliters of lysis solution were added to the control samples, which were then placed at room temperature for 5 min. Then, the cell culture plates were subjected to centrifugation at 600 g for 5 min. After centrifugation, 10 µL of supernatant from each well was transferred to a new 96-well plate and mixed with LDH reaction mixture. After 30 min of sample treatment, the absorbance of the samples was measured using a microplate reader (Multiskan GO, Thermo Fisher Scientific, Waltham, MA, USA) at 450 nm wavelength.

### 2.3. CCK-8 Assay

Cell Counting Kit-8 (CCK-8, Dojindo Laboratories, Kumamoto, Japan) was used to determine the effects of LDC and TTC on cell metabolism. CCD-1064sk cells were placed in 96-well plates at approximately 10,000 cells per well. After the cells reached confluence, CCK-8 solution was added to each well and reacted for 24 h. Each LA was treated for 2 h, and then the absorbance was measured at 450 nm wavelength with a microplate reader (Multiskan GO, Thermo Fisher Scientific, Waltham, MA, USA).

### 2.4. Surface Morphological Analysis

Cell surface morphological changes were observed in the control cell sample and the samples treated with LDC and TTC using field emission scanning electron microscopy (FESEM, S-4300; Hitachi, Tokyo, Japan). Briefly, CCD-1064sk cells were placed in 6-well plates such that each well contained 10,000 cells and incubated for 2~3 days for proliferation. Then, the cell samples were treated with the desired concentrations of LDC and TTC for 12 h.

For FESEM cell sample preparation, the previously described hexamethyldisilane (HMDS) dehydration method was utilized [18]. Briefly, the cells were chemically adhered by using 2% glutaraldehyde (Alfa Aesar, Haverhill, MA, USA) and placed in a fume hood for at least 2 h. The samples were then washed three times with 0.1 M HEPES buffer for 5 min with small agitation. After that, the samples underwent a series dehydration process with ethanol (50%, 70%, 95%, and 100%) for 5 min each. Then, the samples were subjected to a mixture of ethanol and HMDS at ratios of 2:1, 1:1, and 1:2 for 15 min each and finally dried with HMDS (100%) for 10 *h* in a fume hood. The prepared samples were placed on aluminum stubs, sputter coated with platinum using a sputter coater (Q150T; Quorum Technology, Lewes, UK), and analyzed using FESEM.

### 2.5. Colorimetric Assay

Liposomes were prepared using a previously described film hydration method [19,20]. Briefly, 10,12-tricosadiynoic acid (TCDA) (Sigma-Aldrich, St. Louis, MO, USA), a polydiacetylene, was dissolved in chloroform in a glass vial. Then, the chloroform was evaporated by using dry argon (Ar) gas. The dried film was rehydrated with Tris-HCl buffer (0.125 mM, pH 7.6) to achieve a final lipid concentration of 3 mM. The TCDA solution was heated at 80 °C for 30 min in a water bath (WiseBath, WB-6; Daihan, Wonju-si, Korea) and sonicated for 10 min in an ultrasonic processor (Vibra Cell, Sonics VCX -750; Sonics & Materials, Newtown, CT, USA) to achieve a homogenous vesicle solution. After sonication, the solution was filtered with a 0.80 μm syringe filter (dismic-25cs; Advantech, Tokyo, Japan) to remove any large particles and stored at 4 °C overnight. Photoinduced polymerization was carried out by exposing the solution to 254 nm UV light for 2 min, which turned the milky white solution blue.

LDC and TTC were dissolved in DMSO and mixed with TCDA liposome solution to achieve final concentrations of 0.01, 0.05, 0.1, 0.5, 1, 5, 10, 50, and 100 mM. For the control sample, DMSO was mixed with TCDA liposomes to avoid any unwanted negative effects on the liposome sample. The DMSO was kept at a 1% TCDA concentration. After 20 min of reaction at room temperature, the fluorescence spectrum of the liposome solution was measured by a microplate spectrophotometer (PHERAstar FSX Multimode Microplate Reader; BMG Labtech, Ortenberg, Germany). The degree of fluorescence response (FL) was calculated from the equation defined below [21]:

FL = FL_f_ − FL_0_ (1)

where FL_0_ is the initial fluorescence response of vesicles without local anesthetics and FL_f_ is the final fluorescence response of vesicles with local anesthetics.

### 2.6. Data Analysis

The curve fitting and calculation of the IC50 or EC50 values were performed using Origin software (Originlab Corp. Northampton, MA, USA). The sigmoidal curve was fitted by using Boltzmann function:

(2) $$y=\frac{A_{1}-A_{2}}{1+e^{\left(x-x_{0}\right)/\mathrm{dx}}}+A_{2}$$

where A_1_ = initial value, A_2_ = final value, x_0_ = center, dx = time constant, 50% threshold at (x_0_, (A_1_ + A_2_)/2). For statistical analysis we used a simple T test and found that *p* < 0.001 is considered significant.

### 2.7. Electrophysiology Assay with the Model Protein gA

Planar lipid membranes were fabricated using a well-known painting method [22,23]. Briefly, a 3% (*w*/*v*) lipid solution was prepared by dissolving 1,2-diphytanoyl-sn-glycero-3-phosphocholine (DPhPC; Avanti Polar Lipid, Inc., Alabaster, AL, USA) in n-decane. The lipid solution was painted around the aperture (50–100 μm) of a 10 μm thick PTFE film (Good Fellow Cambridge Ltd., Huntingdon, UK). The painted lipid solution was dried at room temperature for approximately 1 h. After drying, the film was sandwiched between two chambers filled with buffer solution. The aperture was then painted again with lipid solution, which resulted in spontaneous formation of a lipid monolayer. The monolayers fused upon the thinning of the droplet, forming a bilayer.

To analyze the ion channel activity in the bilayer, 0.1% gA dissolved in DMSO was used. LLC and TTC were mixed with buffer solution at various concentrations (1, 3, 6 and 10 mM) to analyze their effect on the lipid bilayer membrane and ion channel. Electrical measurements were analyzed using an Axopatch patch amplifier (Molecular Devices, San Jose, CA, USA), and optical bilayer formation was observed with a microscope (Digital Blue, QX5, Marietta, GA, USA). The data sampling rate was recorded at 250 kHz, filtered through a low-pass Bessel filter at 1 kHz and analyzed using the program Clampfit 10.2.

## 3. Results and Discussion

### 3.1. Cell Cytotoxicity Analysis

**Figure 2:**
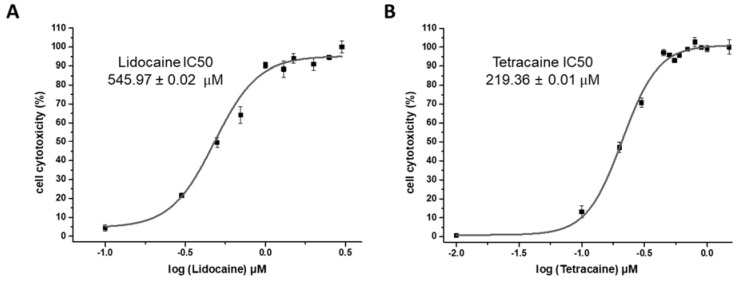
LDH (Lactate Dehydrogenase) assay to analyze cell cytotoxicity, (**A**) Lidocaine IC50 is 545.971 µM (**B**) Tetracaine IC50 is 219.36 µM. (n = 3, *p* < 0.001).

**Figure 3:**
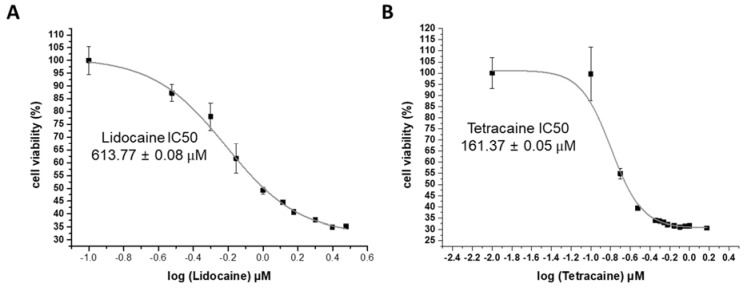
CCK-8 assay to analyze cell cytotoxicity, (**A**) Lidocaine IC50 is 613.77 µM (**B**) Tetracaine IC50 is 161.37 µM. (n = 3, *p* < 0.001).

To quantitatively analyze the cytotoxic effect of the LAs on the cell membrane, an LDH assay was carried out [24]. LDH is an enzyme present in all cell types and is released upon cell membrane damage. The LDH colorimetric assay measures the amount of LDH released in the sample solution through a series of enzymatic reactions. Cell cytotoxicity was measured with regard to LDC concentrations from 0 to 3 mM and TTC concentrations from 0 to 1.5 mM. As shown in Figure 2, the 50% inhibitory concentration (IC50) of LDC was 545.97 ± 0.02 µM, and the IC50 of TTC was 219.36 ± 0.01 µM, indicating that the concentration of TTC IC50 was half that of lidocaine. This result means that TTC has more toxic effects than LDC on the cell membrane. We also measured the changes in the viability of CCD-1064sk cells according to the concentration of LAs using the CCK-8 assay to further confirm their cytotoxicity. As shown in Figure 3, the IC50 of LDC calculated by the CCK-8 assay was 613.77 ± 0.08 μM, whereas the IC50 of TTC was 161.37 ± 0.05 µM, consistent with the results from the LDH assay.

**Figure 4:**
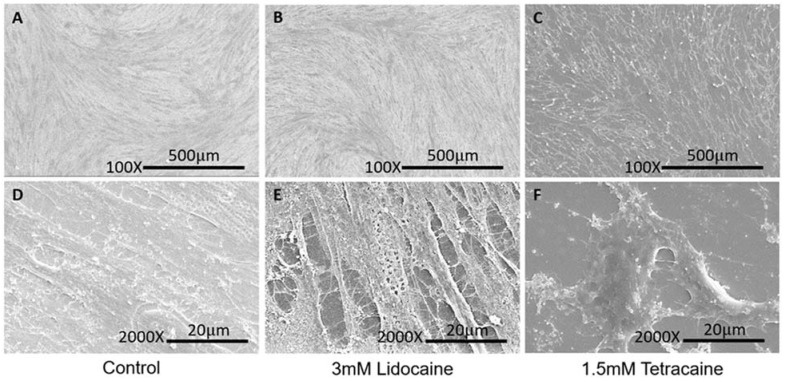
SEM images of CCD-1064sk (human skin fibroblast). (**A**–**C**) Images of CCD-1064sk magnified 100 times in control, 3 mM lidocaine, 1.5 mM tetracaine, respectively. (**D**–**F**) Images of CCD-1064sk magnified 2000 times in control, 3 mM lidocaine, 1.5 mM tetracaine, respectively.

The distribution of LAs on the membrane was observed by FESEM in cell morphological studies. LDC (3 mM) and TTC (1.5 mM) were used to treat CCD-1064sk at the soft tissue level at concentrations that resulted in over 90% cell death in the LDH assay. Under the given concentrations, the images show the most dramatic changes of the cell surfaces. Figure 4 shows FESEM images of the human skin cells. Untreated cells showed no identifiable morphological changes when magnified at 100X and 2000X (Figure 4A,D). However, membrane damage occurred upon treatment with 3 mM LDC, as can be observed in the form of pores and empty spaces in the images. Similarly, 1.5 mM TTC led to cell death, which can be seen in the figures. This result shows that LAs affect the membrane and that TTC is more toxic than LDC. However, comparing the results of the CCK-8 and LDH assays reveals that TTC showed higher toxicity than LDC in the CCK-8 assay because TTC induces apoptosis by affecting mitochondria [25] in addition to causing membrane damage. In Figure 4, only membrane damage was observed for LDC, whereas apoptotic cell rounding and shrinkage were also observed for TTC.

### 3.2. Membrane Affinity Analysis

**Figure 5:**
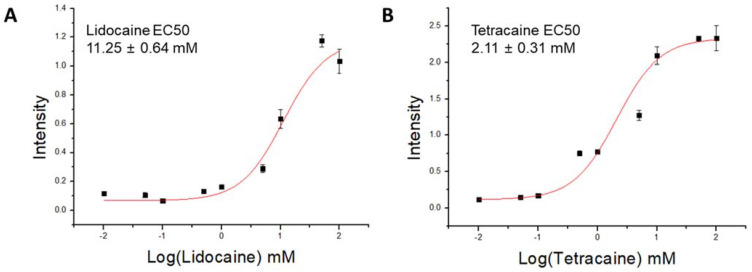
Concentration response curve to calculate EC50 (**A**) The lidocaine EC50 is 11.25 mM (**B**) The tetracaine EC50 is 2.11 mM. (n = 3, *p* < 0.001).

Previously reported studies showed that the partitioning of molecules into lipid membranes can be analyzed using a PDA colorimetric assay [21,26], which is more straightforward than other conventional methods [27]. The effect of local anesthetics can be expected to be substantially consistent with membrane affinity. Therefore, to quantitatively measure the membrane affinity of LAs, TCDA vesicles that have amphiphilic properties similar to those of cell membranes were used. Tetracaine and lidocaine were added at concentrations of 0.01, 0.05, 0.1, 0.5, 1, 5, 10, 50, and 100 mM and reacted at room temperature for 20 min. The degree of color transition was measured by setting the fluorescence emission intensity to 560 nm and the excitation wavelength to 485 nm. The EC50 was then measured using Origin Pro 8.5 with the degree of FL responses with regard to the concentrations, as shown in Figure 5. The calculated EC 50 values for lidocaine and tetracaine were 11.25 ± 0.64 mM and 2.11 ± 0.31 mM, respectively. It is clear from the graph that TTC showed a lower EC50 value than LDC, which means that the membrane affinity of TTC is higher than that of LDC. Although both TTC and LDC have a similar molecular structure, the length of the carbon tail of TTC is longer since it contains a greater number of C-H groups, which ultimately makes it more hydrophobic and hence shows higher membrane affinity. When comparing the results of the IC50 of the LDH assay (Figure 2) and EC50 of the PDA experiment (Figure 5), the toxicity of lidocaine was relatively higher despite its low affinity. This means that lidocaine may have a greater influence on the membrane than tetracaine. To further confirm the interaction between LAs and membranes, indirect studies were further carried out with a model membrane experiment integrated with an ion channel, gA.

### 3.3. Electrophysiological Analysis

**Figure 6:**
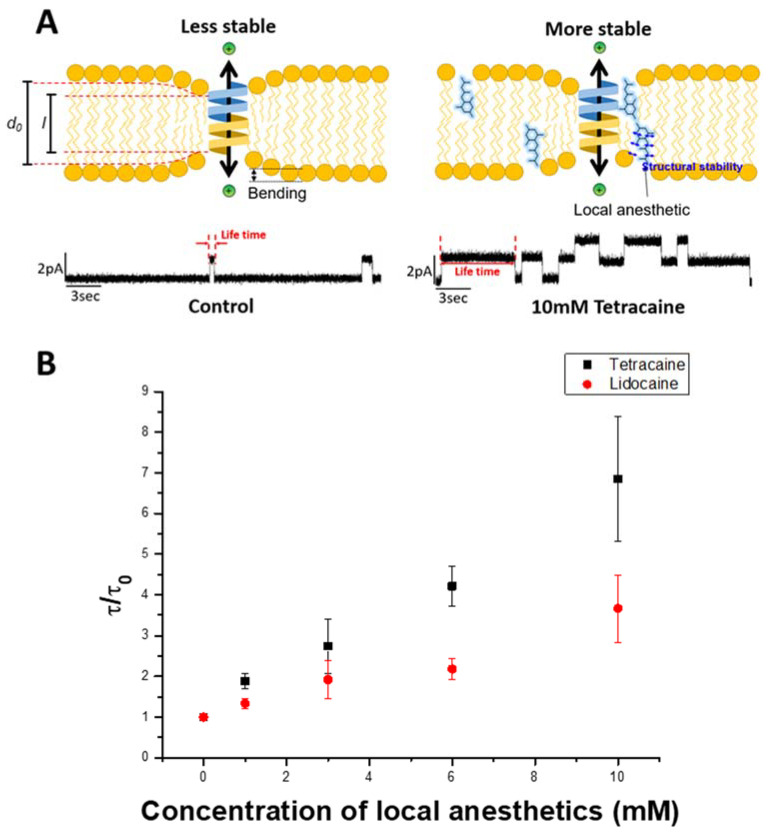
(**A**) The scheme of electrophysiology assay with amplifier. (**B**) Lifetime of gA (gramicidin A) dimer measurement for tetracaine (black dots) and lidocaine (red dots) for 30 min at 20 °C.

A gA channel can be used as a model protein to monitor changes in the structural properties of lipid bilayers [28]. gA is a single-stranded peptide consisting of 15 amino acids that can be purified from *Bacillus brevis*. It forms two right-handed β-helices in hydrophobic membranes. When gA monomers in each leaflet of the bilayer laterally diffuse and combine with each other, they form dimers and allow ions to pass through the gramicidin pore [29,30]. This spontenous dimer formation can be measured using a patch clamp amplifier in realtime. However, the hydrophobic length (l) of the gA dimer channel is approximately 2.2 nm, which is shorter than the typical hydrophobic thickness ($d_{0}$) of the membrane, 3.5 nm [31,32]. As shown in Figure 6A, the channel formation involves surrounding bilayer thickness adjustment. As a result, the bilayer deforamtion energy contributes to the channel formation as the following equation:

(3) $$\Delta G_{\mathrm{total}}=\Delta G_{\mathrm{gA}}+\Delta G_{\mathrm{def}}$$

where $\Delta G_{\mathrm{gA}}$ is the free energy change of the gA channel and $\Delta G_{\mathrm{def}}$ is energy change due to the bilayer deformation [33]. The gA channel behavior is determined by the difference of the elastic energies in the states of the coaxial pair and dimer [34]. However, the deformation energy contributes to the energetic cost of the channel formation, and changes in $\Delta G_{\mathrm{def}}$ are reflected in gA channel behavior such as channel lifetime (τ) or channel appearence rate (f) [35]. The disjoining force (F) of gA dimer, expressed as:

(4) $$F=-\frac{d\left(\Delta G_{\mathrm{def}}\right)}{d\left(l-d_{0}\right)}=2H_{B}\;\left(d_{0}-l\right)+H_{C}C_{0}$$

where $H_{B}$ and $H_{C}$ are the elastic coefficient of bilayer compression and bending moduli, respectively, and $C_{0}$ is the lipid intrinsic curvature. Therefore, F will be decreased in (a) small protein-bilayer mismatch ($d_{0}-l$), (b) small $H_{B}{\;\mathrm{and}\;H}_{C}$ and (c) high value of $C_{0}$. A decrease in F will increase the f and τ [32,33,36].

Any change in the lipid bilayer composition alters the physical properties of the bilayer. As LAs are water-soluble amphiphiles, they infuse into the lipid bilayer and increase its fluidity, which in turn makes $C_{0}$ more positive and hence increases the channel lifetime (τ). To observe the channel lifetime of gA upon the addition of LAs, an electrophysiological method using planar lipid bilayer membranes was used. The effects of LAs were measured at various concentrations (1, 3, 6, and 10 mM) by examining the lifetime of gA dimers. As shown in Figure 6B, τ/τ_0_ (gA dimer lifetime/gA dimer lifetime without LAs) shows an increasing trend with an increasing concentration of LAs. In the case of LDC, the normalized lifetime was 1.33 at 1 mM LDC and 3.66 at 10 mM LDC. TTC also shows a similar effect, with a channel lifetime of 1.88 at 1 mM TTC and 6.85 at 10 mM TTC. The increasing lifetime of the gA channels with increasing concentrations of LAs suggests that LAs affect the bilayer elastic moduli and intrinsic monolayer curvature ($C_{0}$), which results in a decrease in $\Delta G_{\mathrm{def}}$. At a similar concentration, TTC increased the gA channel lifetime (τ/τ_0_) by almost twice as much as LDC, which shows that TTC affects the bilayer elastic moduli and $C_{0}$ of the membrane more.

Our results show that TTC can be easily integrated into the bilayer, thus enhancing its bending around the gA channel due to higher membrane affinity, resulting in a higher curvature structural stability and lower curvature elasticity. The reduction in $\Delta G_{\mathrm{def}}$ is affected not only by how many amphiphiles are integrated into the membrane but also by how much they stabilize the curvature depending on their structure [28]. When gA forms a dimer, the surrounding lipid bilayer bends to form a dense inner layer and a thicker outer layer. Therefore, if the inserted molecule has a more positive cone shape, gA is more stabilized. Moreover, the stabilization of lipid curvature around the gA dimer by LAs is determined by their structural characteristics and membrane affinity. It is assumed that LDC has a more positive cone shape than TTC, which stabilizes the positive curvature [9]. However, LDC has a shorter aromatic ring, which reduces the membrane affinity compared to that of TTC; thus, TTC showed more effects than LDC in the gA experiment. As a result, the higher IC50 value of LDC in the LDH experiment can be attributed to structural factors, while LDC has low membrane affinity. Table 1 summarizes the concentrations of LAs used for different assays (IC50, EC50) and normalized gA lifetime. When LDC and TTC are present at the same concentration in the membrane, LDC causes membrane instability and induces curvature of the membrane more effectively than TTC due to their structural differences. Due to its very low affinity, however, LDC shows lower cell toxicity than TTC because the affinity of the molecules is a more dominant factor than their structural effect.

## 4. Conclusions

In this study, we observed that LAs could perturb cell membranes based on their IC50 value using the LDH assay and CCK-8 assay for CCD-1064sk cells. The factors of LAs that affect the membrane were further confirmed by using a model cell membrane. Through a colorimetric assay, we found that membrane affinity is the most prominent factor that alters the physical properties of membranes. Furthermore, electrophysiological analysis was performed to verify the membrane affinity and structural effects using a model membrane protein. We found that membrane affinity plays a more crucial role in altering cell membrane characteristics than does the structural conformation of the compounds. TTC has a longer hydrocarbon tail than LDC, which increases its hydrophobicity and membrane affinity and hence leads to higher solubility in the membrane. On the other hand, LDC with a short hydrocarbon tail gives a more positive intrinsic curvature of the membrane but partitions into the membrane less, which decreases its structural stability. In short, our results show that the membrane affinity is more dominant than the steric effect when LAs interact with cell membranes. Accordingly, this result helps to understand the mechanism of side effects of LAs, such as cardiotoxic effects [1], and can be used to develop new alternative LAs to minimize side effects.

## Figures and Tables

**Table 1:** Summaries of the LAs concentration employed in IC50 and EC50 analysis, and normalized gA dimer lifetime measurement at 10 mM of LAs.

Local Anesthetics	IC50 LDH Assay	IC50 CCK Assay	EC 50 PDA Assay	Normalized gA Dimer Lifetime at 10 mM
**Lidocaine**	545.97 ± 0.02 μM	613.77 ± 0.08 μM	11.25 ± 0.64 mM	3.66 ± 0.82
**Tetracaine**	219.36 ± 0.01 μM	161.37 ± 0.05 μM	2.11 ± 0.31 mM	6.85 ± 1.53

## Data Availability

Not applicable.
